# Swimming as a Positive Moderator of Cognitive Aging: A Cross-Sectional Study with a Multitask Approach

**DOI:** 10.1155/2012/273185

**Published:** 2012-12-26

**Authors:** Amira Abou-Dest, Cédric T. Albinet, Geoffroy Boucard, Michel Audiffren

**Affiliations:** UMR CNRS 7295, University of Poitiers, Sport Sciences Faculty, Bât. A5, 5 rue Théodore Lefebvre, 86000 Poitiers, France

## Abstract

This study examined whether regular swimming in older adults was related to better cognitive functioning and whether there were any global or selective positive effects of this physical activity (PA) on cognition. The cognitive performances of three groups of sixteen volunteer participants (young adults, sedentary older adults, and older adults who regularly practice swimming) were evaluated using a multitask approach. All participants performed a battery of ten tasks: two reaction time tasks assessing information processing speed and eight experimental tasks assessing three executive functions (EFs), (behavioral inhibition, working memory updating, and cognitive flexibility). The results showed that young adults performed significantly better than older adults on all examined cognitive functions. However, in older adults, regular swimming was related to better performance on the three EFs, but not on information processing speed. More precisely, five experimental tasks out of the eight tapping EFs were shown to be sensitive to positive effects from swimming practice. Finally, the demonstrated benefits of swimming on EFs were not necessarily linked to better cardiorespiratory fitness. The present findings illustrate the validity of using a multitask approach in examining the potential benefits of regular PA on cognitive aging.

## 1. Introduction

The growth of the proportion of the population aged 65 and older in the industrialized world, as well as in developing countries, has profound implications for public health and the economic costs of medical care. Cerebral and cognitive decline, as a function of aging, represents a predominant cause of autonomy loss in aging populations. According to the prefrontal-executive theory [1], executive functions (EFs) and their underpinning prefrontal and frontal brain structures are particularly sensitive to the effects of normal and pathological aging [2, 3]. Therefore, the preservation of these brain areas and their associated cognitive functions is of particular importance. Chronic physical activity (PA), aimed at improving cardiorespiratory health, has been proposed to be a good, practical, and powerful candidate to overcome cerebral and behavioral declines [4–6]. Accordingly, the principal aim of the present study was to examine the potential benefits of an understudied form of PA—regular swimming (one of the most popular and accessible forms of PA for older adults)—on EF performance in a population aged 65 to 80 years old.

Executive functions involve higher-order functions of control and coordination allowing behavioral adaptation to complex or novel situations for which automatisms or routines are inappropriate. As such, EFs refer to a set of cognitive processes involved in goal and strategy formulation, planning and monitoring, mental flexibility, and behavioral inhibition [7, 8]. This multicomponent or fractionated view implies that EFs may be composed of different executive processes that are at least partially independent, yet are sufficiently correlated to represent a unique construct. In this line, an authoritative model was developed by Miyake and colleagues [9], who proposed that EFs can be fractionated into at least three separable processes; cognitive flexibility (shifting between multiple tasks or mental sets), behavioral inhibition (suppression of dominant, automatic, or prepotent responses when necessary), and updating of working memory (substitution of old information by new more relevant information in working memory). This three-factor structure of EFs has been frequently postulated in the literature and replicated in older populations [2, 10, 11]. Although caution is still needed when choosing the tasks used to assess this construct, all three EFs have been quite constantly shown to be impaired in older people, even after having controlled for other confounders such as level of education and information processing speed [2]. 

In contrast to the prefrontal-executive theory, the processing speed theory [12] assumes that age-related cognitive declines are accounted for by generalized slowing of cognitive processing due to a diffuse or global deterioration of white matter integrity throughout the whole brain with aging. A meta-analysis by Colcombe and Kramer [13] showed that the effect size of the positive effects of chronic exercise is significantly smaller on information processing speed than on executive functions. It is then very interesting to compare the effects of aging and physical activity on these two functions in the same experiment.

In contrast to this negative view of cognitive aging, a new line of research has developed works that examine how keeping a physically active lifestyle can maintain or even improve cognition and brain functions. In recent years, using various methodologies, several studies have shown that older adults who maintain a physically active way of life by participating in regular PA or chronic exercise outperform their sedentary counterparts in cognitive performance, exhibit higher brain plasticity [14, 15] and are more efficiently protected against neurological diseases and dementia [16, 17]. Moreover, randomized controlled trials have demonstrated that short physical training programs (from 3 months to 1 year) improve cognitive performance, particularly EFs, in sedentary older adults with no pathology of the central nervous system [18–21] or dementia [22, 23]. Narrative and meta-analytic reviews tend to show that this positive effect may be particularly selective to attentional, controlled cognitive functions involving EFs (see [4, 13, 24]) and that the PA programs should involve a strong proportion of aerobic exercises (see [4, 13, 25]). Moderate-to-vigorous PA induces improvements in cardiorespiratory fitness (indexed by maximal oxygen uptake, VO_2_max) and leads to a cascade of neurophysiological mechanisms such as the release of neurotrophic factors that facilitate neurogenesis and/or angiogenesis [19, 26–28], two mechanisms known to be associated with higher brain plasticity, which ultimately leads to improvements in cognitive performance. It is important to note that, on the one hand, the putative links between neurophysiological mechanisms and efficiency of cognitive processes are not yet fully understood and, on the other hand, the possible mechanisms explaining these links are most likely not exclusive. A number of reviews on this topic suggest a concomitant increase in cardiorespiratory fitness, brain plasticity, and cognitive performance, but inconsistent results have been reported concerning the last point. For instance, some authors have been unable to show cognitive improvements after an aerobic exercise program that was sufficient to increase VO_2_max [29]. Others have shown that not all EFs are enhanced after a PA program and that the improvement in cognitive performance, when effective, was not related to the improvement in cardiorespiratory fitness [21, 25]. Finally, Liu-Ambrose et al. [30] recently reported the results of a randomized controlled study showing that a 12-month resistance training program (involving strength exercises twice a week) induced the same improvements in EF performance and functional plasticity as an aerobic program. These findings emphasize the need to further study the relationships between PA and cognitive performance in the aged population. In the present study, we were particularly interested in one understudied aerobic physical activity: swimming. To the best of our knowledge, only one study was designated to explicitly test the specific relationship between water aerobics and cognitive improvements in older adults [31]. In this study, Hawkins and collaborators examined the influence of a 10-week program of basic swimming skills on variations of two experimental tasks involving processing speed, attentional switching, and dual-task time-sharing. They found that subjects who trained in water aerobics showed significantly larger improvements on the executive control tasks than a nontrained control group. Interestingly, similar performance improvements were demonstrated for both groups in the nonexecutive tasks. Thus, it is important to verify whether this isolated finding can be generalized and to determine if swimming benefits are global or selective to some executive processes given the fractionated reality of EFs. To that end, we employed a clear theoretically driven framework to examine the potential mediator role of VO_2_max in this relationship.

The objectives of the present study were twofold. First, we wanted to replicate our team's previous results on age-related declines in EF and processing speed performance [2] by comparing the performances of younger and older adults on various tasks that assess information processing speed and the three postulated EFs, inhibition, working memory updating, and shifting. Second, assuming these age-related cognitive declines, we wanted to determine the selectivity of the relationship between swimming and the three EFs and information processing speed, in our sample of older adults. An important feature of this experiment was to combine the use of two or three cognitive tasks that are well-known for tapping each of the cognitive functions of interest with a multivariate approach. This procedure was shown to accurately assess the construct of EF and to reveal age-related executive declines in a previous pool of participants [2]. Swimming was chosen for three main reasons. (1) It is an understudied aerobic activity in the field of cognitive aging, and it solicits all the muscle groups and increases cardiorespiratory fitness. (2) It is one of the most accessible and practiced PAs in the elderly and is rated as the second most popular PA in France for the age range 65 and older [32]. (3) It is less traumatic on joints than walking or jogging. Several reviews have described the beneficial effects of water-based exercise on physical fitness parameters, such as aerobic capacity and strength in the elderly [33–35]. The elderly take a particular interest in water activity because it reduces the fear of falling, limits tolerance for weight-bearing activities, and enhances adherence and participation [36].

## 2. Methods

### 2.1. Participants

Thirty-two older adults aged 65 to 80 years (16 sedentary people and 16 swimmers) and 16 younger adults (18–30 years), all of whom were free of neurological and cardiovascular disease, participated to this study. The older participants were recruited from senior community centers, civic groups, and aquatic centers through the use of flyers and newspapers. All the older participants were screened by their personal physicians who rated them as being in good health and signed a medical certificate indicating no contraindications for cardiorespiratory fitness testing. Younger participants were recruited from the University of Poitiers. 

Inclusion criteria for older adults were as follows: (a) being aged between 65 and 80 years; (b) having adequate mental status as indicated by a score strictly greater than 25 on the Mini Mental State Examination (MMSE) [37]; (c) for the sedentary group, leading a sedentary lifestyle with no participation in any structured PA as assessed by a validated PA questionnaire, the Dijon Score of Physical Activity (DSPA) [38]; (d) for the active group, leading a physically active lifestyle as assessed first by a history of regularly swimming at least twice a week for at least two years but no other regular PA. Secondly, the DSPA was also administered for the active group to ensure a significant difference in the amount of physical activity between the two groups. Inclusion criteria for younger adults were being aged from 18 and 30 years. The exclusion criteria were the following: (a) using medication that could affect cardiovascular health or cognitive functions; (b) cardiorespiratory or neurological disease; (c) major surgery within one year prior to testing. All participants gave written informed consent and the study was approved by the local ethics committee. The demographic and physical characteristics of all participant groups are displayed in Table 1. The active older adults (swimmers) had been practicing swimming for two to 43 years (M = 2.56 years; SD = 0.79), two to five times a week. The mean session duration was between 45 and 100 minutes (M = 64.38 min; SD = 15.37). 

### 2.2. Evaluation of Cardiorespiratory Fitness

For the older participants, VO_2_max was estimated by the Rockport Fitness Walking test [39]. This submaximal field test has been shown to accurately estimate VO_2_max in populations similar to the one in the present study [39, 40]. Participants were required to walk one mile (1609 m) as quickly as possible, and heart rate was continuously recorded by a Polar RS 800 beat-to-beat recorder (Polar Electro, Oy, Kempele, Finland). VO_2_max was calculated using the equation developed by Kline and collaborators.

### 2.3. Evaluation of Information Processing Speed

Information processing speed was measured through two reaction time tasks: an auditory simple reaction time (SRT) task and a visual 2-Choice Reaction Time (CRT) task [2]. SRT and CRT were used as the main dependent variables and the error rate in the CRT task enabled checking for possible speed-accuracy tradeoffs.

### 2.4. Evaluation of Executive Functions

Each of the three EFs was assessed via 2 or 3 different experimental tasks. All tasks were the same as those used in a previous study from our laboratory that involved a different sample of younger and older adults [2]. We refer the reader to this paper for a full description of the procedure and tasks used. 

#### 2.4.1. Inhibition

**The Stroop Task**

In this computerized version of the Stroop task, the dependent variable was the difference in mean RTs (ms) between incongruent trials (e.g., the word RED written in blue) and neutral trials (e.g., XXXXX written in blue) for correct responses. The error rate in incongruent and neutral conditions was also controlled to check for any possible speed-accuracy tradeoffs.

**The Stop-Signal Task**

In this task, participants were required to respond as quickly as possible to a visual stimulus by pressing the corresponding key, unless an auditory tone rang out requiring to abort (or inhibit) the prepotent motor response. The dependent variable selected for the multivariate analyses of variance (MANOVAs) was the rate of successful inhibition calculated as the probability (*P*, between 0 and 1) of successful inhibition. The stop-signal reaction time (SSRT) has also been calculated according to Logan's race model [41]. This variable was not included in the MANOVAs for two reasons: (1) SSRT correlated moderately with SRT and CRT (*r* = .41 and .50, resp.), while *P*(*I*) did not (see Table 2); (2) SSRT did not correlate with the interference score measured in the Stroop task and the adjacency score measured in the RNG task, two variables reflecting inhibition (*r* = .13 and *r* = −.06, resp.), while *P*(*I*) correlated significantly with the adjacency score (see Table 2).

**The Random Number Generation (RNG) Task**

In this task, participants were required to generate and say aloud series of random numbers from one to nine at the rate of one digit per second. The dependent variable that reflected inhibition for this task was the Adjacency score (A in %), ranging from 0% (no neighboring pairs, i.e., good performance) to 100% (only neighboring pairs, i.e., poor performance). The RNG task necessitates inhibiting counting in ascendant or descendant series, and the Adjacency score measures the tendency of the participants to count by one.

#### 2.4.2. Updating

**The Verbal Running Span Task**

In this computerized task, participants were required to recall serially the last four letters of a list of six, eight, ten, or twelve consonants that were presented visually. The dependent variable was the number of letters correctly recalled in the right order (max. = 48).

**The spatial Running Span Task**

Like for the previous task, in this computerized task, participants were required to recall serially the last four spatial locations of a dot in a 4 × 4 matrix. The dependent variable was the number of dots correctly recalled in the right order (max. = 48).

#### 2.4.3. Shifting

**The Dimension-Switching Task**

In this computerized task, participants watched on the computer screen the French words for LEFT or RIGHT enclosed in a left or right arrow, and displayed above or below the center of the white screen. Depending on instructions, they were required to respond to the word or to the direction of the arrow by pressing the correct corresponding key. The dependent variable selected for this task was the global switch cost for correct responses, calculated as the difference in RT (ms) between trials from the simple blocks and task-repeat trials from the mixed blocks. The error rate in simple and mixed blocks of trials was calculated to check for any possible speed-accuracy tradeoffs. The local switch cost was also calculated but was not included for further analyses because it did not correlate with the number of perseverative errors in the Wisconsin Card Sorting Task (*r* = .18) while the global switch cost did (*r* = .32, see Table 2).

**Stimulus-Response Compatibility Switching Task**

In this computerized task, participants watched an arrow pointing left or right, surrounded by a frame on the computer screen. Depending on the color of the frame, participants were to press the response key located either on the side pointed by the arrow (green or blue frame), or on the opposite side (red or orange frame). The dependent measure for this task was the local switch cost for correct responses, calculated as the difference in RT (ms) between task-repeat trials and task-switch trials during the mixed blocks. The error rate for repetition and alternation trials was also computed to check for any possible speed-accuracy tradeoffs. The global switch cost has not been computed in this task because the number of trials in the simple blocks was too small; the simple blocks have only been used to familiarize participants to the rules of mapping between stimuli and responses.

**The Wisconsin Card Sorting Test (WCST)**

In this computerized version of the WCST, participants were required to sort a set of cards according to three different rules that changed periodically. The dependent variable that reflected shifting for this task was the number of perseverative errors.

For the processing speed construct and for each EF component, a standardized Cronbach alpha was computed to assess how well the selected tasks measured a latent construct. Alphas for each postulated cognitive function were low to good (standardized Cronbach alphas were .77, .60, .78, and .59 for Information processing speed, Inhibition, Updating, and Shifting, resp.). Table 2 presents the results of the bivariate correlations between all cognitive tasks within the whole sample.

### 2.5. Procedure

After careful screening for inclusion and exclusion criteria, older participants were first evaluated for cardiorespiratory fitness on one day. Second, each participant was tested individually in a quiet experimental room in two sessions on different days separated by a minimum of two days and a maximum of seven days. Each session lasted approximately 1.5–2 hours. The two sessions were counterbalanced across participants and, within each session, all tasks were counterbalanced across participants. Participants were given short breaks between each experimental task. 

### 2.6. Statistical Analyses

The data on cognitive performance were analyzed in three ways. First, a series of one-way multivariate planned analyses of covariance (MANCOVAs) was conducted on each set of dependent variables reflecting a specific cognitive function (speed of information processing, behavioral inhibition, updating of working memory, and shifting), contrasting young adults and seniors with level of education (number of years) as a covariate. This first series of planned MANCOVAs was conducted in order to test the effect of age on each cognitive function. The level of education was entered as a covariate because younger adults showed a higher level of education than older adults (see Table 1). When the effect of age on a cognitive function was significant, we conducted a series of planned analyses of covariance (ANCOVAs) on each cognitive function task, contrasting young adults and seniors with level of education as a covariate. Second, a series of one-way multivariate planned analyses of variance (MANOVAs) was conducted on the same sets of dependent variables only in older adults, contrasting regular swimmers and sedentary people. This second series of planned MANOVAs was conducted in order to test the effect of physical activity level on each cognitive function. When the result of the MANOVA was significant, we performed a series of planned analyses of variance (ANOVAs) on each of the individual tasks composing cognitive function. Finally, for the older adult sample, simple regression analyses were performed between VO_2_max and EF scores when there was a significant correlation between these variables. The level of significance was set at *P* < .05 and Cohen's *d* was reported for pairwise comparisons as a measure of effect size. As proposed by Cohen [42], a “small” effect is when *d* = 0.2, a “medium” effect when *d* = 0.5, and a “large” effect when *d* = 0.8. For the MANOVAs and MANCOVAs, partial eta squared (Partial *η* ^2^) was also reported as a measure of the percentage (when multiplied by 100) of variance explained by the treatment factors.

## 3. Results

### 3.1. Group Differences in Demographics and Global Cognition

As seen in Table 1, young adults attended more years of education than older participants (*F*(1, 46) = 12.97; *P* < 0.05). In addition, as expected, older active participants were more physically active than their sedentary counterparts as revealed by a higher score on the DPAS (*F*(1, 30) = 60.75; *P* < 0.05), and a higher level of VO_2_max (*F*(1, 29) = 5.13; *P* < 0.05), but the two groups did not differ in MMSE scores.

### 3.2. Effects of Chronological Age on Cognitive Functions

Results of the statistical analyses and behavioral performance as a function of age group for each cognitive function and each cognitive task are detailed in Table 3. As seen, there was a significant effect of age on each cognitive function, even after having controlled for the level of education. Subsequent ANCOVAs on each individual dependent measure revealed that the age-related effect was significant for each experimental task (see Table 3) except for three: the stop-signal task, the letter running-span, and the WCST, for which there was no significant difference between younger and older adults after having controlled for education level.

The effect of age was also examined on error rate for all the tasks that included choice reaction time measurements. A first ANOVA was conducted on error rate in the CRT task with age as between-subjects factor. There was no significant effect of age (*F*(1, 45) = 0.22; *P* > .63); error rate = 2.81% and 2.44% for younger and older adults, respectively. A second ANOVA with age as between-subjects factor and type of trials (neutral versus incongruent) as within-subjects factor was then conducted on error rate in the Stroop task. The interaction between age and type of trials was close to significance (*F*(1, 45) = 3.40; *P* > .07), the simple effect of age was not significant (*F*(1, 45) = 0.98; *P* > .32) while the simple effect of type of trials reached significance (*F*(1, 45) = 12.10; *P* < .002). Participants made significantly more errors in the incongruent condition (2.43%) than in the neutral condition (0.35%). A third ANOVA with age as between-subjects factor and type of block of trials (simple versus mixed) as within-subjects factor was conducted on error rate in the dimension-switching task. The interaction between age and type of block of trials was close to significance (*F*(1, 45) = 3.60; *P* > .06), as well as the simple effect of age (*F*(1, 45) = 3.18; *P* > .08), but the effect of type of block of trials reached significance (*F*(1, 45) = 26.25; *P* < .0001). Participants made significantly more errors in the mixed block of trials (5.07%) than in the simple block of trials (1.61%). A fourth and last ANOVA with age as between-subjects factor and type of trials (alternation versus repetition) as within-subjects factor was conducted on error rate in the mixed blocks of trials of the stimulus-response compatibility switching task. The interaction between age and type of transition did not reach significance (*F*(1, 45) = 1.89; *P* > .17), but the simple effects of age and type of transition did (*F*(1, 45) = 6.07; *P* < .02 and *F*(1, 45) = 37.10; *P* < .0001, resp.). All participants made more errors when they had to alternate (9.27%) rather than when they had to repeat the stimulus-response mapping (3.33%) and older adults (7.63%) made more errors than younger adults (3.63%). These results on error rate and those concerning RTs (see Table 3) showed that there was no speed-accuracy tradeoffs for the effect of age on cognitive functions assessed through RT tasks. 

### 3.3. Effects of Swimming Practice on Cognitive Functions Performance in Older Adults

The results of the statistical analyses and behavioral performance as a function of level of activity for each cognitive function and each experimental task are detailed in Table 4. The MANOVAs revealed a significant effect of swimming activity on each of the three EF components but no significant effect on information processing speed. Subsequent ANOVAs on each task tapping EFs showed that older participants who swim regularly performed significantly better in five out of eight tests. More precisely, swimmers outperformed sedentary participants on the two tasks tapping Updating, on two of three tasks tapping Shifting, and on the RNG task tapping Inhibition. When it was significant, the effect size of swimming was large (mean Cohen's *d* = 1.05). 

The effect of swimming was examined on error rate for all the tasks that included choice reaction time measurements. A first ANOVA was conducted on error rate in the CRT task with PA level as between-subjects factor. There was a significant effect of PA level on error rate (*F*(1, 45) = 8.24; *P* < .007); sedentary older adults made more errors in the CRT task (3.75%) than older swimmers (1.13%). A second ANOVA with PA level as between-subjects factor and type of trials (neutral versus incongruent) as within-subjects factor was conducted on error rate in the Stroop task. There was a significant interaction between these two factors (*F*(1, 45) = 8.07; *P* < .007). A post hoc test showed that sedentary older adults made more errors in the incongruent condition than in the neutral condition (3.39% versus 0%, respectively; *P* < .02) while older adults who practiced swimming did not (1.24% versus 0.26% resp.; *P* > .45). A third ANOVA with PA level as between-subjects factor and type of block of trials (simple versus mixed) as within-subjects factor was conducted on error rate in the dimension-switching task. The interaction between PA level and the type of block of trials was significant (*F*(1, 45) = 10.81; *P* = .002). A post hoc test showed that older swimmers made more errors in the mixed than in the simple block of trials (8.68% versus 1.61%; *P* < .0002) while sedentary older adults did not (3.26% versus 1.67%; *P* > .34). The examination of RT and error data in the dimension-switching task suggests that the two groups of older adults did not use the same strategy in the more difficult condition: swimmers preferred to emphasize speed whereas sedentary people preferred to emphasize accuracy. A fourth and last ANOVA with PA level as between-subjects factor and type of trials (alternation versus repetition) as within-subjects factor was conducted on error rate in the mixed blocks of trials of the stimulus-response compatibility switching task. The interaction between PA level and type of transition did not reach significance (*F*(1, 45) = 0.61; *P* > .43), but the simple effects of PA level and type of transition did (*F*(1, 45) = 12.43; *P* < .001 and *F*(1, 45) = 33.25; *P* < .0001, resp.). Older adults made more errors when they had to alternate (11.07%) rather than when they had to repeat the stimulus-response mapping (4.19%) and, overall, older swimmers (10.94%) made more errors than sedentary older adults (4.33%). One more time, swimmers preferred to emphasize speed while sedentary participants preferred to emphasize accuracy in the more difficult condition. 

### 3.4. Relationship between Cardiorespiratory Fitness and Executive Function

**Figure 1:**
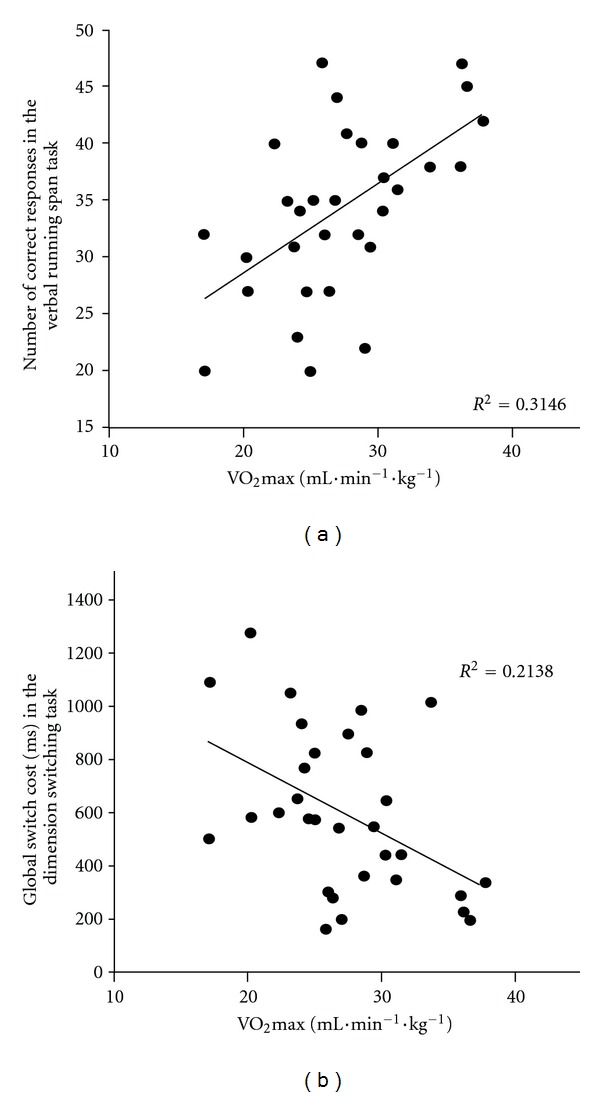
Scatter plots relating VO_2_max level and updating performance of verbal information (a) and global switch cost (b) in the older participants.

To examine the relationships between cardiorespiratory fitness and EF performance in older adults, we performed a series of bivariate correlations (Spearman coefficients of correlation) between each cognitive task performance and VO_2_max level. The performance of only two tasks significantly correlated with VO_2_max level: the updating of verbal information (*r* = .56; *P* < .05) and the global switch cost (*r* = .46; *P* < .05).  Figure 1 depicts the scatter plots of executive performance on these tasks with VO_2_max level with their respective coefficients of determination (*R* ^2^). 

## 4. Discussion

As has been extensively documented, age-related cerebral and cognitive declines are not uniform but quite differentiated (see [43, 44] for reviews). Similarly, most of the recent literature on aging, fitness, and cognition has shown that chronic exercise would result in selective improvements in executive functions rather than general benefits [13, 20, 21]. However, contemporary theoretical models of executive functions posit that the construct of EF is not uniform but encompasses fractionated executive subprocesses [2, 8, 9]. It is then important to examine whether chronic exercise benefits are homogeneous or selective to some particular executive subprocesses. Using this established theoretical and methodological framework, the aim of this study was to examine differences in cognitive performance as a function of chronological age and long-term regular practice of swimming by measuring younger and older adults' information processing speed and performance on three well-known EFs, inhibition, updating, and shifting. Globally, the results revealed a clear pattern of age-related decline in all cognitive functions. On the other hand, older adults who practice regular swimming were shown to have a positive relation with improved performance on five of eight experimental tasks and all EF components. VO_2_max level was only positively related to executive performance for two tasks. These results provide a clear argument for the use of a multitask approach when studying the effects of chronic exercise on cognitive functions.

Before examining the effects of aging and swimming practice on cognitive performance, it is important to discuss the multitask approach used in this study. Because it is impossible to find a “pure” task to assess an isolated cognitive function, particularly an EF, multiple measures were used to rule out “task impurity” (see [2, 7–9]). As such, we wanted to assess the EF construct at the level of a general or latent variable, which reflects the postulated cognitive function more strongly than each individual experimental task alone. However, as revealed by the standardized Cronbach alphas and the matrix of correlations displayed in Table 2, it appears that the construct validity of our three EFs was quite low. Indeed, the correlations between tasks supposed to reflect a particular EF component were often low or even nonsignificant and sometimes smaller than the correlations between two tasks thought to assess different EF components. Such results have already been reported by previous studies from our own team [2] and others [11, 45] and raise a question of the low specificity of the tasks used to assess EFs, which will be discussed in the limitations section.

Consistent with a large body of research on cognitive and cerebral aging, we found that chronological age has a detrimental effect on information processing speed and on the three evaluated EFs. The age-related decline in the RT tasks is in agreement with numerous studies on the effect of aging on processing speed [2, 12]. The performance deficits observed in our older adults in the three EF domains also agree with the literature examining this question with a similar approach [2, 10, 11]. Our results also suggest that the deleterious effects of aging are more pronounced for processing speed (69% of the explained variance, see partial *η* ^2^ in Table 3) than for updating (48% of the explained variance), shifting (37% of the explained variance), and inhibition (23% of the explained variance). However, it is important to note that taking into account the educational level (as measured by the number of years of academic education) decreased the age-related effects on all the cognitive tasks and even eliminated the effects of chronological age on the letter running-span task and the WCST. This finding strengthens the importance of taking into account education as an important moderator of cognitive aging which can be interpreted in the more general context of cognitive reserve [46, 47]. As we will discuss below, it may be that PA (in the present study, swimming) could also be viewed as a proxy of cognitive reserve beyond educational level.

In the older population, the differences in cognitive performance between swimmers and sedentary people were not homogeneous across all the studied cognitive processes. First, there was no significant difference between active and sedentary participants in the measures of information processing speed, as assessed by the SRT and the CRT tasks (see Table 4). This result contrasts with some previous results [48, 49] but agrees with others [20, 21, 24], particularly with those of Hawkins and coworkers [31] in the same context of swimming practice. These contradicting findings may be due to specific differences between particular PAs practiced by different samples of participants. The impact on processing speed may be different between racket sports or hand-ball, such as in Spirduso's study [49], and swimming such as in our study. Another, more likely possibility is linked to the assertion that chronic exercise exerts selective effects on cognitive processes involving executive control and not on less-controlled processes, such as the stimulus-driven information processing involved in basic reaction time tasks (see [13, 20, 24]). Our results favor this second possibility and strengthen this selective benefit of PA on EF processes. 

Second, our results indicate prima facie that the impact of swimming practice was quite homogeneous across all three EF components, assuming that our groups only differed at the level of swimming practice. All three MANOVAs contrasting the performance between older swimmers and sedentary participants were significant for these cognitive functions. However, the percentage of variance explained by swimming practice was more important for Shifting (42%) than for Updating (30%) and Inhibition (16%), as revealed by partial eta squared (see partial *η* ^2^ in Table 4). Our results of a strong relationship between swimming practice and Shifting confirm the previous conclusions of Hawkins et al. [31] on the positive effect of water aerobics on attentional flexibility. However, as pointed out in the Results section, the examination of speed and accuracy measures in the two switching tasks underscores that the two groups of participants may not have used the same strategies to perform these tasks. The swimmers made more errors and emphasized speed to the detriment of accuracy. Consequently, the speed-accuracy tradeoffs observed in the two switching tasks weaken the strength of the positive relationship between swimming practice and Shifting.

Moreover, five out of eight tasks were strongly sensitive to differences between swimmers and sedentary participants, but the performance of two tasks out of three (Stroop task and signal-stop task) assessing inhibition and one task out of three (dimension-switching task) assessing shifting did not significantly differ between regular swimmers and sedentary participants. As previously stated, we think that this result underscores the importance of using a multitask approach when studying the effects of chronic exercise on cognitive functions and may help to resolve some discrepant results in this domain. For example, the absence of significant effect of exercise training reported by other researchers on some cognitive tasks [21, 29, 50] may not reflect an absence of real effects but instead a lack of sensitivity of the experimental tasks. For instance, our results conflict with those reported by Smiley-Oyen et al. [21], because they found an effect of aerobic exercise on the Stroop task and no effect on the WCST. Several reasons can be hypothesized to explain the discrepancies, such as differences in study design, experimental tasks, modes of response, and selected dependent measures, but it is always difficult to definitively conclude which is the correct explanation. One of the strengths of the present study is to combine the use of several different experimental tasks to examine the influence of PA within the same sample not only at the level of individual tasks but also at the more general level of the explored cognitive functions. This procedure allowed us to show that regular swimming was clearly related to better executive performance on the three examined EFs, despite the absence of significant effect on three of the eight tasks. This may indicate that some tasks could be more sensitive to the effects of PA than other tasks. Additional work should verify the consistency of our results, as our findings are far from conclusive. A recent study from our group [51] used different measures of PA and explored the same executive functions with different tasks or variations of the same tasks as used in the present study and showed that PA exerted a selective effect on inhibition but not on updating or shifting and only for the oldest adults of the sample (71 years and older). These different results highlight the lability of the effects of exercise on cognitive performance according to tasks and characteristics of the participants. Clearly, more work is needed to draw a definitive conclusion on the selectivity of the effects of chronic exercise effects on EFs.

The role of cardiorespiratory fitness in the relationship between PA and cognitive performance in older adults remains under debate (see [4, 21, 25]). Accordingly, we were interested in examining the relationships between VO_2_max level and cognitive performance. As shown in Figure 1, only performance on the verbal running span task and the dimension-switching task was significantly correlated with VO_2_max level. Of particular interest, one must remember that there was no significant effect of PA on the global switch cost of the dimension-switching task (*P* = .11, see Table 4). Thus, there is only one task (the verbal running span task) showing PA-related benefits that could be mediated by cardiorespiratory fitness level and one task (the dimension-switching task) showing no relationship with swimming practice but a significant relationship with VO_2_max level. These results underscore the differences between a behavioral measure assessed through questionnaires (PA) and an estimated physiological measure assessed through field test (VO_2_max), and their different influences on different cognitive functions [21, 25, 51]. Distinguishing between PA and cardiorespiratory fitness, which were weakly related (*r* = .35; *P* = .054) in the present study, seems of particular importance because they may exert their influence on cognitive functioning by different mechanisms. A challenge for future studies will be to understand and characterize the mechanisms underlying these selective effects. One may hypothesize that PA could be viewed qualitatively as a proxy of cognitive reserve, as defined by Stern and collaborators [46, 47], allowing physically active older adults to make flexible and efficient use of available brain reserve and to demonstrate better cognitive performance. In other respects, better cardiorespiratory fitness could induce anatomical and neurophysiological changes in the brain [6, 14, 26–28] that could reflect and enhance brain reserve [46, 47]. 

 There are potential limitations in the present study that should be addressed. First, the low specificity of the tasks used to assess the construct of EF in this study limits the generalizability of our conclusions and strengthens the importance of looking for and using well-designed tasks to reflect the postulated cognitive functions. Second, our cross-sectional design precludes inferences about causality in the relationship between chronic engagement in swimming and executive performance. Well-designed randomized-controlled trials are now needed, using the same multitask approach, before a definitive conclusion can be made. Third, the level of education of the participants has been taken into account as a covariate in the statistical analyses of the present study. However, other important moderators of aging related to lifestyle, such as income, socioeconomic status, food habits, previous careers, and lifelong leisure activities, have not been measured in our experiment. These different variables can explain part of the variance in cognitive aging and may lead to an overestimation of the relationship between present level of physical activity and cognitive performance in cross-sectional designs, as could have been the case in the present study.

To summarize the principal findings, this study demonstrated the validity of using a multitask approach in examining the potential benefits of regular swimming on the aging of cognitive function. Such a theoretical and methodological approach allowed us to show that chronic swimming practice is related to better executive functions in older adults and that these benefits are seen on the three EFs studied but that some tasks are less sensitive to detecting these benefits. Finally, the demonstrated benefits of PA were not necessarily linked to better cardiorespiratory fitness, showing the potential relative independence of these behavioral or physiological moderators on executive performance.

## Figures and Tables

**Table 1:** Characteristics of the three groups of participants.

Characteristics	Young adults	Active older adults	Sedentary older adults	Young versus older adults	Active versus Sedentary
*n*	16	16	16	*P* = 1	*P* = 1
Gender M/F	9/7	9/7	9/7	*P* = 1	*P* = 1
Age (years)	23.56 (2.56)	69.13 (3.88)	69.25 (3.25)	*P* < .05	*P* = .92
MMSE (max = 30)	29.31 (0.01)	28.75 (0.93)	28.69 (1.08)	*P* = .06	*P* = .86
Education (years)	16.09 (1.25)	13.75 (3.11)	13.56 (1.93)	*P* < .05	*P* = .84
DPAS (max = 30)	—	24.25 (1.48)	15.17 (4.37)	—	*P* < .05
VO_2_max (mL/min/kg)	—	29.35 (5.13)	25.27 (4.94)	—	*P* < .05

Note. M: Male, F: Female, MMSE: Mini Mental State Examination, DPAS: Dijon Physical Activity Score.

**Table 2:** Matrix of correlations between the indices of cognitive performance, one index per cognitive task, for the whole sample.

Dependent variable/Task	1	2	3	4	5	6	7	8	9	10
(1) Auditory SRT	1.00									
(2) Visual CRT	.63*	1.00								
(3) *P*(*I*)	−.05	−.02	1.00							
(4) Interference cost	.42*	.43*	**.18**	1.00						
(5) Adjacency score	.16	.34*	.31*	.51*	1.00					
(6) NO correct letters	−.18	−.29*	.10	−.26	−.56*	1.00				
(7) NO correct locations	−.30*	−.69*	−.03	−.51*	−.63*	.64*	1.00			
(8) NO perseverative errors	.23	.30*	.04	.40*	.58*	−.52*	−.55*	1.00		
(9) Global switching cost	.08	.54*	−.03	.20	.49*	−.51*	−.69*	.32*	1.00	
(10) Local switching cost	.25	.46*	.01	.20	.46*	−.40*	−.44*	**.22**	.42*	1.00

Note. SRT: Simple reaction time, CRT: Choice reaction time, *P*(*I*): Rate of successful inhibitions, *: *P* < .05, bold: Correlations within the same cognitive function.

**Table 3:** Results of the MANCOVAs, ANCOVAs, and effect-sizes contrasting young and older participants on cognitive performance, with level of education as covariate, and mean behavioral performance for each age group (SD).

Cognitive functions	Cognitive tasks	Dependent variables	MANCOVA F	ddl	Wilk's *λ*	Partial *η* ^²^	ANCOVA F(1,44)	Cohen's *d *	Young adults M (SD)	Older adults M (SD)
	**Auditory simple reaction time task**	**Reaction time (ms)**					**23.06** (*P * ** < .0001**)	**1.51**	**194 (26)**	**257 (52)**
**Speed of information processing**			**47.98** (*P * ** < .0001**)	**2, 43**	**.31**	**.69**				
	**Visual choice reaction time task**	**Reaction time (ms)**					**94.89** (*P * ** < .0001**)	**3.34**	**335 (28)**	**475 (53)**

	Stop-signal task	Rate of successful inhibition					1.13 (*P* = .29)	0.16	0.41 (0.15)	0.44 (0.21)
**Behavioral inhibition**	**Stroop task**	**Interference cost (ms)**	**4.29** (*P * ** < .001**)	**3, 42**	**.77**	**.23**	**12.46** (*P * ** < .001**)	**1.10**	**189 (63)**	**291 (115)**
	**RNG task**	**Adjacency score**					**5.96** (*P * ** < .02**)	**0.93**	**33.50 (7.04)**	**42.27 (11.29)**

	Letter running-span task	NO correct responses					2.69 (*P* = .11)	0.80	39.19 (4.62)	34.28 (7.35)
**Updating of working memory**			**19.77** (*P * ** < .0001**)	**2, 43**	**.52**	**.48**				
	**Spatial running-span task**	**NO correct responses**					**36.81** (*P * ** < .0001**)	**2.23**	**41.25 (4.84)**	**23.31 (10.30)**

	Wisconsin card sorting test	NO perseverative errors					3.69 (*P* = .06)	0.79	8.50 (2.68)	12.81 (7.22)
**Shifting**	**Dimension switching task**	**Global switch cost (ms)**	**8.37** (*P * ** < .0002**)	**3, 42**	**.63**	**.37**	**15.65** (*P * ** < .0003**)	**1.74**	**192 (154)**	**606 (299)**
	**Stimulus-Response switching task**	**Local switch cost (ms)**					**9.86** (*P * ** < .0031**)	**1.09**	**71 (57)**	**184 (135)**

Note. RNG: Random Number Generation, in bold: Significant effect.

**Table 4:** Results of the MANOVAs, ANOVAs, and effect-sizes contrasting active and sedentary older participants on cognitive performance and mean behavioral performance for each age group (SD).

Cognitive function	Cognitive task	Dependent variable	MANOVA F	ddl	Wilk's *λ*	Partial *η*²	ANOVA F(1,45)	Cohen's *d *	Sedentary older adults M (SD)	Active older adults M (SD)
	Auditory simple reaction time task	Reaction time (ms)					—	—	252 (40)	262 (64)
Speed of information processing			0.43 (*P* = .73)	2, 44	.98	.02				
	Visual choice reaction time task	Reaction time (ms)					—	—	468 (49)	482 (57)

	Stop-signal task	Rate of successful inhibition					0.00 (*P* = .96)	0.02	0.44 (0.23)	0.44 (0.20)
**Behavioral inhibition**	Stroop task	Interference cost (ms)	**2.79** (*P*=** .05**)	**3, 43**	**.84**	**.16**	0.10 (*P* = .76)	0.10	297 (132)	285 (99)
	**RNG task**	**Adjacency score**					**7.04** (*P * ** < .011**)	**0.85**	**46.72 (11.09)**	**37.82 (9.91)**

	**Letter running-span task**	**NO correct responses**					**19.50** (*P * ** < .0001**)	**1.45**	**29.94 (6.08)**	**38.63 (5.88)**
**Updating of working memory**			**9.61** (*P * ** < .0004**)	**2, 44**	**.70**	**.30**				
	**Spatial running-span task**	**NO correct responses**					**7.04** (*P * ** < .013**)	**0.80**	**19.44 (8.39)**	**27.19 (10.80)**

	**Wisconsin card sorting test**	**NO perseverative errors**					**21.70** (*P * ** < .0001**)	**1.41**	**17.00 (8.05)**	**8.63 (2.36)**
**Shifting**	Dimension switching task	Global switch cost (ms)	**10.24** (*P * ** < .0001**)	**3, 43**	**.58**	**.42**	2.65 (*P* = .11)	0.68	704 (267)	508 (306)
	**Stimulus-Response switching task**	**Local switch cost (ms)**					**6.24** (*P * ** < .017**)	**0.76**	**232 (129)**	**135 (126)**

Note. RNG: Random Number Generation, in Bold: Significant effect.
